# Expeditious Synthesis of Dianionic-Headed 4-Sulfoalkanoic Acid Surfactants

**DOI:** 10.3390/molecules22040640

**Published:** 2017-04-16

**Authors:** Jianhui Jiang, Jiaxi Xu

**Affiliations:** 1 State Key Laboratory of Chemical Resource Engineering, Department of Organic Chemistry, Faculty of Science, Beijing University of Chemical Technology, Beijing 100029, China; xjjjh78@163.com; 2 Engineering Laboratory of Chemical Resources Utilization in South Xinjiang of Xinjiang Production and Construction Corps, College of Life Sciences, Tarim University, Alar, Xinjiang 843300, China

**Keywords:** alkanoic acid, double head, oxidation, radical reaction, surfactant, xanthate

## Abstract

4-Sulfoalkanoic acids are a class of important dianionic-headed surfactants. Various 4-sulfoalkanoic acids with straight C8, C10, C12, C14, C16, and C18 chains were synthesized expeditiously through the radical addition of methyl 2-((ethoxycarbonothioyl)thio)acetate to linear terminal olefins and subsequent oxidation with peroxyformic acid. This is a useful and convenient strategy for the synthesis of dianionic-headed surfactants with a carboxylic acid and sulfonic acid functionalities in the head group region.

## 1. Introduction

**Figure 1:**
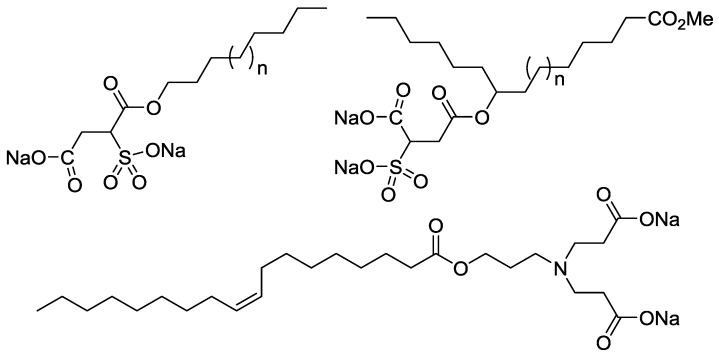
Some reported dianionic-headed surfactants.

**Scheme 1:**
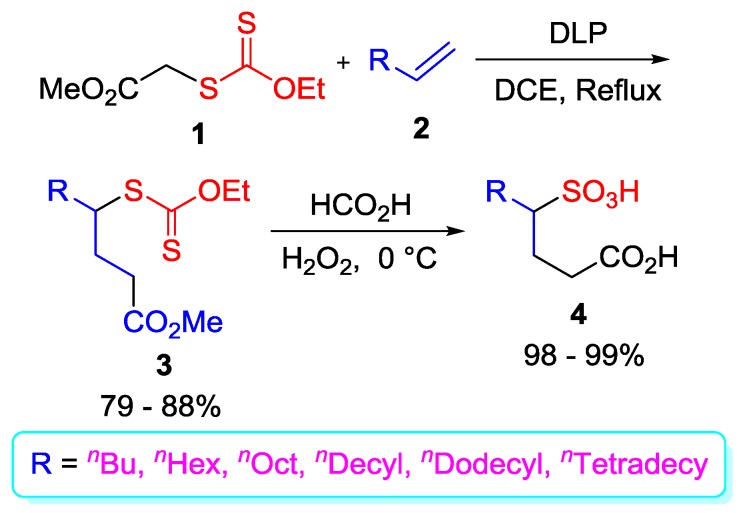
Synthesis of dianionic-headed 4-sulfoalkanoic acid surfactants.

Surfactants have been widely applied in almost every fields, including personal care and industry [1]. Numerous gemini surfactants have been prepared and investigated during the last several decades [2,3]. Recently, much attention has been paid to the preparation and properties of double-headed and double-tailed surfactants [4,5,6,7]. Only a few double-tailed surfactants have been prepared, and their surfactant activity has not been evaluated until now [4,5]. Double-headed surfactants have been utilized in the industry as wetting agents and dispersants (Figure 1) [6,7]. They have been generally prepared from maleic anhydride and maleate-monoester/diesters [8,9]. There is considerable and still increasing interest in the synthesis of new double-headed surfactants with two different dianionic heads, because dianionic-headed surfactants with two hydrophilic head groups and one hydrophobic tail with a head to tail ratio of 2:1 generally show good wetting and low foam properties alongside mild surface activity. They may find applications in the textile industry [7] and colloidal drug delivery system [10]. Zard’s xanthate radical addition chemistry promotes us to develop a new strategy to synthesize a series of novel dianionic-headed surfactants with a carboxylic acid and sulfonic acid functionalities in the head group region [11,12,13,14,15]. Herein, we present an expeditious synthesis of dianionic-headed surfactant 4-sulfoalkanoic acids through the radical addition of methyl 2-((ethoxycarbonothioyl)thio)acetate to linear terminal olefins and subsequent oxidation with peroxyformic acid (Scheme 1).

## 2. Results and Discussion

**Scheme 2:**
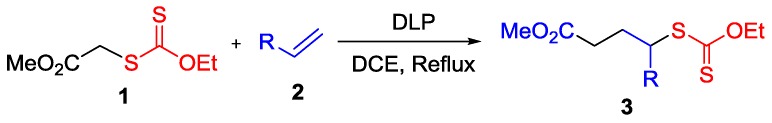
Synthesis of xanthates **3**.

Methyl 2-((ethoxycarbonothioyl)thio)acetate **1** was prepared from potassium *O*-ethylxanthate and methyl chloroacetate [16]. Reactions of methyl 2-((ethoxycarbonothioyl)thio)acetate **1** and linear terminal olefins **2**, including 1-hexene, 1-octene, 1-decene, 1-dodecene, 1-tetradecene, and 1-hexadecene, under radical initiator dilauroyl peroxide (DLP) in 1,2-dichloroethane (DCE) as a solvent gave rise to a series of xanthates, methyl 2-((ethoxycarbonothioyl)thio)alkanoates **3** in good to excellent yields (Scheme 2 and Table 1) (Supplementary materials) [17].

**Scheme 3:**
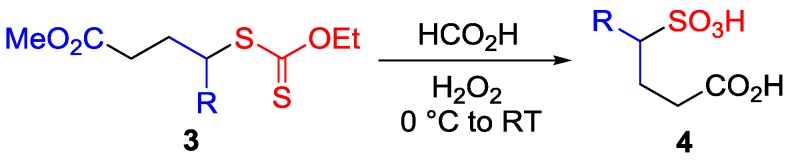
Synthesis of 4-Sulfoalkanoic acids **4**.

Through the previously mentioned oxidation procedure with peroxyformic acid [18,19,20], all xanthates **3** were converted into the corresponding 4-sulfoalkanoic acids **4** in almost quantitative yields. Under the current acidic oxidation conditions, the xanthate group was oxidized into sulfonic acid and the methyl-carboxylate group in xanthates **3** was hydrolyzed into the carboxylic acid group (Supplementary materials) (Table 2 and Scheme 3).

The designed synthetic strategy shows excellent efficiency with the following advantages; simple and inexpensive starting materials, a two-step synthetic route, good to excellent yields, and easy purification in the last step.

**Scheme 4:**
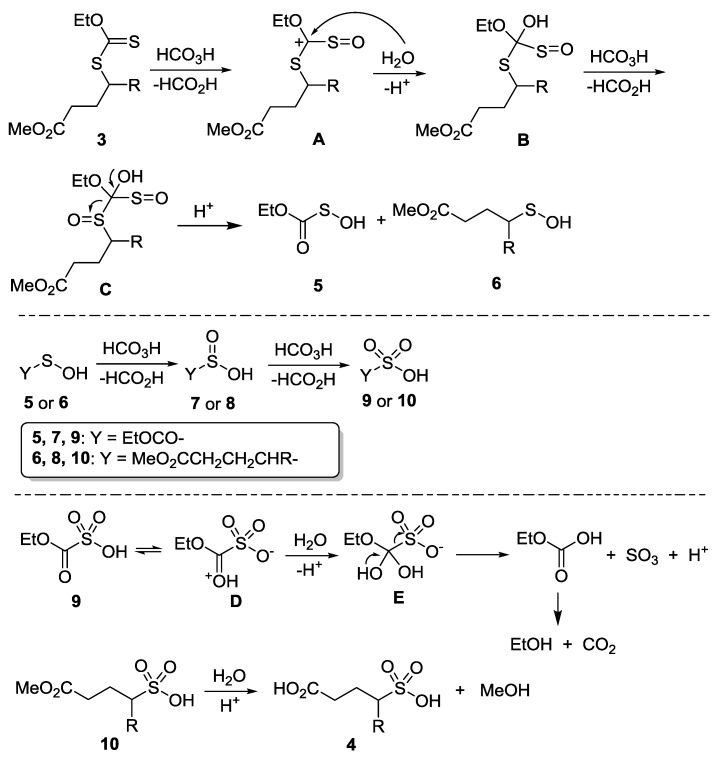
Plausible mechanism for the oxidation of methyl 2-((ethoxycarbonothioyl)thio)alkanoates **3** to 4-sulfoalkanoic acids **4**.

We previously prepared taurine and homotaurine derivatives by oxidation of thioacetates [21,22,23,24,25] and xanthates [18,19,20]. Douglass and his coworkers reported that xanthates (ROCS_2_R’) were chlorinated into alkoxydichloromethanesulfenyl chlorides (ROCCl_2_SCl) and alkylsulfur trichlorides (R’SCl_3_) with chlorine under anhydrous conditions [26,27]. On the basis of above results and our recent results of the oxidative chlorination [28], the mechanism of the oxidation of xanthates **3** into 4-sulfoalkanoic acids **4** with peroxyformic acid was proposed, as shown in Scheme 4. Initially, the sulfur atom in the thioxo group of xanthates **3** is oxidized with peroxyformic acid, generating intermediates **A**. Intermediates **A** are attacked by water in the reaction system to generate intermediates **B**, of which the sulfur atom in their thioether part is further oxidized by another molecule of peroxyformic acid to produce intermediates **C**. Unstable intermediates **C** decompose into ethoxycarbonylsulfenic acid (**5**) and 1-(3-methoxy-3-oxopropyl)alkanesulfenic acids **6** under acidic conditions.

Both ethoxycarbonylsulfenic acid **5** and 1-(3-methoxy-3-oxopropyl)alkanesulfenic acids **6** are further oxidized into the corresponding sulfonic acids **9** and **10**, respectively, with peroxyformic acid following the same mechanism.

Unstable ethoxycarbonylsulfonic acid **9** tautomerizes into intermediate **D**, in which its carbonyl group is protonated by dissociated sulfonic acid. Intermediate **D** is attacked by water, giving rise to intermediate **E**, which is more unstable and finally decomposes into ethanol, CO_2_, SO_3_, and proton. 1-(3-Methoxy-3-oxopropyl)alkanesulfonic acids **9** are further hydrolyzed into 4-sulfoalkanoic acids **4** under acidic conditions (Scheme 4).

## 3. Materials and Methods

### 3.1. Materials and Instruments

Melting points were measured on a Yanaco MP-500 melting point apparatus (Yanaco Ltd., Osaka, Japan) and are uncorrected. ^1^H-NMR and ^13^C-NMR spectra were recorded with a Bruker 400 spectrometer (Bruker Company, Billerica, MA, USA) in CDCl_3_ with tetramethylsilane (TMS) as an internal standard, or in D_2_O with DOH as an internal standard in ^1^H-NMR, or with HCO_2_H (166.3 ppm) as an internal standard in ^13^C-NMR. IR spectra were obtained on a Nicolet AVATAR 330 FTIR spectrometer (Thermo Nicolet Corporation, Madison, WI, USA). HRMS spectra were recorded with a Liquid Chromatography/Mass Spectrometry/Data and Time-of-Flight (LC/MSD TOF) mass spectrometer (Agilent, Santa Clara, CA, USA). TLC analysis was performed on glass pre-coated silica gel YT257‒85 (10‒40 µm) plate (Qingdao Ocean Chemical Industry, Qingdao, China). Spots were visualized with UV light or iodine. Column chromatography was performed on silica gel zcx II (200‒300 mesh) (Qingdao Ocean Chemical Industry, Qingdao, China) with petroleum-ether (PE) and ethyl-acetate (EA) (Beijing Chemical Reagent Company, Beijing, China) as the eluent.

### 3.2. Synthesis of Methyl 2-((Ethoxycarbonothioyl)thio)acetate *(**1**) [16,17]*

To a solution of methyl-chloroacetate (4.175 g, 25 mmol) in acetone (40 mL) precooled at 0 °C, potassium-*O*-ethyl-dithiocarbonate (4.232 g, 27 mmol) was added portionwise while stirring at 0 °C. After the addition, the mixture was allowed to warm to room temperature under continuous stirring. After the removal of acetone, the residue was dissolved in water (50 mL) and the mixture was extracted with CH_2_Cl_2_ (3×50 mL). The combined organic phase was dried over MgSO_4_. After the removal of solvents, the residue was purified on a silica gel column with petroleum ether and ethyl acetate (15:1, v/v) as the eluent to afford the desired xanthate **1**, 4.032 g (83% yield). Its analytic data are identical to the reported ones.

### 3.3 General Procedure for the Synthesis of Methyl-2-((ethoxycarbonothioyl)thio)alkanoates ***3***

A stirred solution of olefin **2** (8 mmol) and methyl 2-((ethoxycarbonothioyl)thio)acetate (**1**) (1.554 g, 8 mmol) in 1,2-dichloroethane (12 mL) was heated at reflux for 15 min. dilauroyl peroxide (DLP) (168 mg, 5 mol %) was added and additional DLP (168 mg, 5 mol %) was added each hour until the methyl 2-((ethoxycarbonothioyl)thio)acetate (**1**) was consumed completely (generally 3 h). The mixture was allowed to cool to room temperature. After the solvent was evaporated under reduced pressure, the residue was purified by flash chromatography on silica gel with a mixture of petroleum-ether and ethyl-acetate (40:1, *v*/*v*) as the eluent to afford the desired product **3**.

#### 3.3.1. Methyl 2-((ethoxycarbonothioyl)thio)octanoate (**3a**)

Yellow oil; yield: 1.756 g (79%). ^1^H-NMR (400 MHz, CDCl_3_): *δ* = 0.90 (t, *J* = 7.2 Hz, 3H, CH_3_), 1.25−1.40 (m, 4H, 2CH_2_), 1.42 (t, *J* = 7.1 Hz, 3H, CH_3_), 1.66 (q, *J* = 7.3 Hz, 2H, CH_2_), 1.86−1.96 (m, 1H in CH_2_), 2.06−2.15 (m, 1H in CH_2_), 2.47 (dt, *J* = 1.4, 7.4 Hz, 2H, CH_2_), 3.68 (s, 3H, CH_3_), 3.76 (quint, *J* = 6.8 Hz, 1H, CH), 4.64 (q, *J* = 7.2 Hz, 2H, CH_2_). ^13^C-NMRNMR (101 MHz, CDCl_3_): *δ* = 13.8, 13.9, 22.5, 28.9, 29.5, 31.4, 34.0, 50.7, 51.7, 69.8, 173.5, 214.4. IR (KBr): 2955.3, 2929.3, 2857.9, 1739.9, 1436.8, 1212.5, 1111.4, 1050.1. cm^-1^ HRMS (ESI): *m/z* calcd for C_12_H_23_O_3_S_2_^+^: 279.1083 [M + H]^+^; found: 279.1080.

#### 3.3.2. Methyl-2-((ethoxycarbonothioyl)thio)decanoate (**3b**)

Yellow oil; yield: 2.105 g (86%). ^1^H-NMR (400 MHz, CDCl_3_): *δ* = 0.88 (t, *J* = 7.0 Hz, 3H, CH_3_), 1.25−1.35 (m, 7H, 3CH_2_ & 1H in CH_2_), 1.42 (t, *J* = 7.1 Hz, 3H, CH_3_), 1.36−1.48 (m, 1H in CH_2_), 1.66 (q, *J* = 7.1 Hz, 2H, CH_2_), 1.86−1.96 (m, 1H in CH_2_), 2.05−2.15 (m, 1H in CH_2_), 2.48 (dt, *J* = 1.4, 7.0 Hz, 2H, CH_2_), 3.68 (s, 3H, CH_3_), 3.76 (quint, *J* = 6.8, 1H, CH), 4.64 (q, *J* = 7.2 Hz, 2H, CH_2_). ^13^C-NMRNMR (101 MHz, CDCl_3_): *δ* = 13.8, 14.0, 22.6, 26.7, 29.1, 29.5, 31.4, 31.6, 34.3, 50.8, 51.7, 69.8, 173.5, 214.4. IR (KBr): 2953.6, 2927.2, 2855.8, 1740.2, 1436.5, 1365.8, 1212.8, 1111.3, 1050.6 cm^−1^. HRMS (ESI): *m/z* calcd for C_14_H_27_O_3_S_2_^+^: 307.1396 [M + H]^+^; found: 307.1391.

#### 3.3.3. Methyl-2-((ethoxycarbonothioyl)thio)dodecanoate (**3c**)

Yellow oil; yield: 2.348 g (88%). ^1^H-NMR (400 MHz, CDCl_3_): *δ* = 0.88 (t, *J* = 7.0 Hz, 3H, CH_3_), 1.23−1.30 (m, 11H, 5CH_2_ & 1H in CH_2_), 1.42 (t, *J* = 7.1 Hz, 3H, CH_3_), 1.35−1.45(m, 1H in CH_2_), 1.67 (q, *J* = 7.1 Hz, 2H, CH_2_), 1.86−1.96 (m, 1H in CH_2_), 2.06−2.14 (m, 1H in CH_2_), 2.40−2.53 (m, 2H, CH_2_), 3.67 (s, 3H, CH_3_), 3.75 (quint, *J* = 6.8 Hz, 1H, CH), 4.64 (q, *J* = 7.1 Hz, 2H, CH_2_). ^13^C-NMRNMR (101 MHz, CDCl_3_): *δ* = 13.8, 14.1, 22.6, 26.8, 29.2, 29.4(2C), 29.5, 31.3, 31.8, 34.3, 50.8, 51.6, 69.8, 173.5, 214.4. IR (KBr): 2951.9, 2925.6, 2854.4, 1740.6, 1436.5, 1365.6, 1212.6, 1111.4, 1051.3 cm^−1^. HRMS (ESI): *m/z* calcd for C_16_H_31_O_3_S_2_^+^: 335.1709 [M + H]^+^; found: 335.1702.

#### 3.3.4. Methyl-2-((ethoxycarbonothioyl)thio)tetradecanoate (**3d**)

Yellow oil; yield: 2.491 g (86%). ^1^H-NMR (400 MHz, CDCl_3_): *δ* = 0.88 (t, *J* = 7.0 Hz, 3H, CH_3_), 1.23−1.28 (m, 15H, 7CH_2_& 1H in CH_2_), 1.42 (t, *J* = 7.1 Hz, 3H, CH_3_), 1.39−1.44 (m, 1H in CH_2_), 1.65 (q, *J* = 7.0 Hz, 2H, CH_2_), 1.86−1.96 (m, 1H in CH_2_), 2.06−2.14 (m, 1H in CH_2_), 2.40−2.53 (m, 2H, CH_2_), 3.67 (s, 3H, CH_3_), 3.72−3.79 (m, 1H, CH), 4.64 (q, *J* = 7.1 Hz, 2H, CH_2_). ^13^C-NMR (101 MHz, CDCl_3_): *δ* = 13.7, 14.1, 22.6, 26.8, 29.27, 29.37, 29.41, 29.45, 29.52, 29.54, 31.3, 31.9, 34.2, 50.8, 51.6, 69.8, 173.5, 214.4. IR (KBr): 2924.2, 2853.9, 1740.5, 1436.7, 1365.5, 1212.5, 1111.4, 1051.6 cm^−1^. HRMS (ESI): *m/z* calcd for C_18_H_35_O_3_S_2_^+^: 363.2022 [M + H]^+^; found: 363.2016.

#### 3.3.5. Methyl-2-((ethoxycarbonothioyl)thio)hexadecanoate (**3e**)

Yellow oil; yield: 2.498 g (80%). ^1^H-NMR (400 MHz, CDCl_3_): *δ* = 0.88 (t, *J* = 7.0 Hz, 3H, CH_3_), 1.23−1.28 (m, 19H, 9CH_2_ & 1H in CH_2_), 1.42 (t, *J* = 7.1 Hz, 3H, CH_3_), 1.39−1.44 (m, 1H in CH_2_), 1.65 (q, *J* = 6.9 Hz, 2H, CH_2_), 1.86−1.96 (m, 1H in CH_2_), 2.06−2.14 (m, 1H in CH_2_), 2.40−2.53 (m, 2H, CH_2_), 3.67 (s, 3H, CH_3_), 3.72−3.79 (m, 1H, CH), 4.64 (q, *J* = 7.1 Hz, 2H, CH_2_). ^13^C-NMRNMR (101 MHz, CDCl_3_): *δ* = 13.8, 14.1, 22.7, 26.8, 29.32, 29.38, 29.42, 29.45, 29.53, 29.59, 29.60, 29.62, 31.3, 31.9, 34.3, 50.8, 51.6, 69.8, 173.4, 214.4. IR (KBr): 2923.5, 2852.9, 1739.9, 1436.1, 1365.9, 1211.9, 1111.3, 1050.3 cm^−1^. HRMS (ESI): *m/z* calcd for C_20_H_39_O_3_S_2_^+^: 391.2335 [M + H]^+^; found: 391.2330.

#### 3.3.6. Methyl-2-((ethoxycarbonothioyl)thio)octadecanoate (**3f**)

Yellow oil; yield: 2.917 g (87%). ^1^H-NMR (400 MHz, CDCl_3_): *δ* = 0.89 (t, *J* = 6.9 Hz, 3H, CH_3_), 1.24−1.35 (m, 23H, 11CH_2_ & 1H in CH_2_), 1.43 (t, *J* = 7.0 Hz, 3H, CH_3_), 1.41−1.47(m, 1H in CH_2_), 1.67 (q, *J* = 7.3 Hz, 2H, CH_2_), 1.88−1.97 (m, 1H in CH_2_), 2.07−2.16 (m, 1H in CH_2_), 2.42−2.54 (m, 2H, CH_2_), 3.69 (s, 3H, CH_3_), 3.77 (quint, *J* = 6.7, 1H, CH), 4.64 (q, *J* = 7.0 Hz, 2H, CH_2_). ^13^C-NMRNMR (101 MHz, CDCl_3_): *δ* = 13.8, 14.1, 22.7, 26.8, 29.36, 29.42, 29.45, 29.46, 29.49, 29.57, 29.63, 29.66, 29.68, 29.69, 31.4, 31.9, 34.3, 50.8, 51.6, 69.8, 173.4, 214.4. IR (KBr): 2924.0, 2853.1, 1741.1, 1436.5, 1366.0, 1211.8, 1111.4, 1051.4 cm^−1^. HRMS (ESI): *m/z* calcd for C_22_H_43_O_3_S_2_^+^: 419.2648 [M + H]^+^; found: 419.2641.

### 3.4. General procedure for the synthesis of 4-sulfonylalkanoic acids **4**

To a stirred and mixed solution of 98% formic acid (15 mL) and 30% H_2_O_2_ (10 mL), xanthate **3** (3 mmol) was added dropwise. The reaction mixture was stirred at room temperature for 2 h and then at 65 °C overnight. The removal of solvents in a vacuum afforded 4-sulfoalkanoic acid **4**.

#### 3.4.1. 4-Sulfooctanoic acid (**4a**)

Yellow oil; yield: 666 mg (99%). ^1^H-NMR (400 MHz, D_2_O): *δ* = 0.53 (t, *J* = 7.2 Hz, 3H, CH_3_), 0.91−1.03 (m, 2H, CH_2_), 1.03−1.13 (m, 2H, CH_2_), 1.13−1.25 (m, 1H in CH_2_), 1.41−1.56 (m, 1H in CH_2_), 1.56−1.70 (m, 1H in CH_2_), 1.70−1.75 (m, 1H in CH_2_), 2.21−2.30 (m, 2H, CH_2_), 2.45−2.50 (m, 1H, CH). ^13^C-NMR (101 MHz, D_2_O): *δ* = 13.8, 21.6, 25.2, 29.0, 29.5, 31.9, 59.7, 178.5. IR (KBr): 2925.9, 2855.2, 1738.9, 1228.2, 1168.4, 1077.7, 1053.6 cm^−1^. HRMS (ESI): *m/z* calcd for C_8_H_15_O_5_S^−^: 223.0646 [M − H]^−^; found: 223.0643.

#### 3.4.2. 4-Sulfodecanoic acid (**4b**)

Colorless oil; yield: 742 mg (98%). ^1^H-NMR (400 MHz, D_2_O): *δ* = 0.45 (t, *J* = 6.4 Hz, 3H, CH_3_), 0.80−0.95 (m, 6H, 3CH_2_), 0.96−1.07 (m, 2H, CH_2_), 1.08−1.22 (m, 1H in CH_2_), 1.41−1.50 (m, 1H in CH_2_), 1.51−1.60 (m, 1H in CH_2_), 1.60−1.70 (m, 1H in CH_2_), 2.13−2.23 (m, 2H, CH_2_), 2.34−2.45 (m, 1H, CH). ^13^C-NMR (101 MHz, D_2_O): *δ* = 14.3, 22.8, 25.2, 27.1, 29.3, 29.9, 31.8, 31.9, 59.7, 178.2. IR (KBr): 2927.5, 2856.9, 1712.2, 1230.1, 1169.1, 1078.4, 1054.2 cm^−1^. HRMS (ESI): *m/z* calcd for C_10_H_19_O_5_S^−^: 251.0959 [M − H]^−^; found: 251.0954.

#### 3.4.3. 4-Sulfododecanoic acid (**4c**)

Colorless oil; yield: 832 mg (99%). ^1^H-NMR (400 MHz, D_2_O): *δ* = 0.40 (t, *J* = 6.4 Hz, 3H, CH_3_), 0.75−0.98 (m, 12H, 6CH_2_), 1.00−1.11 (m, 1H in CH_2_), 1.35−1.51 (m, 2H, CH_2_), 1.51−1.60 (m, 1H in CH_2_), 2.04−2.20 (m, 2H, CH_2_), 2.25−2.37 (m, 1H, CH). ^13^C-NMR (101 MHz, D_2_O): *δ* = 14.5, 23.2, 25.2, 27.6, 29.9, 30.0, 30.1. 30.3, 31.0, 32.5, 59.8, 177.8. IR (KBr): 2926.0, 2855.8, 1709.8, 1230.5, 1169.4, 1053.9 cm^−1^. HRMS (ESI): *m/z* calcd for C_12_H_23_O_5_S^−^: 279.1272 [M − H]^−^; found: 279.1270.

#### 3.4.4. 4-Sulfotetradecanoic acid (**4d**)

Colorless oil; yield: 916 mg (99%). ^1^H-NMR (400 MHz, D_2_O): *δ* = 0.44 (t, *J* = 6.5 Hz, 3H, CH_3_), 0.83−1.05 (m, 16H, 8CH_2_), 1.14−1.20 (m, 1H in CH_2_), 1.39−1.55 (m, 2H, CH_2_), 1.55−1.65 (m, 1H in CH_2_), 2.10−2.21 (m, 2H, CH_2_), 2.33−2.44 (m, 1H, CH). ^13^C-NMR (101 MHz, D_2_O): *δ* = 14.5, 23.3, 25.2, 27.7, 30.16, 30.19, 30.4. 30.5, 30.66, 30.70, 31.0, 31.8, 59.8, 177.7. IR (KBr): 2924.5, 2854.1, 1710.9, 1231.5, 1170.0, 1077.8, 1054.2 cm^−1^. HRMS (ESI): *m/z* calcd for C_14_H_27_O_5_S^−^: 307.1585 [M − H]^−^; found: 307.1581.

#### 3.4.5. 4-Sulfohexadecanoic acid (**4e**)

Colorless oil; yield: 989 mg (98%). ^1^H-NMR (400 MHz, D_2_O): *δ* = 0.58 (t, *J* = 6.4 Hz, 3H, CH_3_), 0.90−1.07 (m, 20H, 10CH_2_), 1.16−1.28 (m, 1H in CH_2_), 1.50−1.71 (m, 2H, CH_2_), 1.71−1.76 (m, 1H in CH_2_), 2.20−2.35 (m, 2H, CH_2_), 2.43−2.53 (m, 1H, CH). ^13^C-NMR (101 MHz, D_2_O): *δ* = 14.5, 23.3, 25.2, 27.8, 29.4, 29.6, 29.7, 29.8. 29.9, 30.2, 30.4, 30.5, 30.6, 30.7, 31.1, 31.9, 59.8, 177.7. IR (KBr): 2924.6, 2853.9, 1710.5, 1288.0, 1069.2, 1011.8 cm^−1^. HRMS (ESI): *m/z* calcd for C_16_H_31_O_5_S^−^: 335.1898 [M − H]^−^; found: 335.1892.

#### 3.4.6. 4-Sulfooctadecanoic acid (**4f**)

Colorless oil; yield: 1.083 g (99%). ^1^H-NMR (400 MHz, D_2_O): *δ* = 0.75 (t, *J* = 7.2 Hz, 3H, CH_3_), 1.07−1.27 (m, 24H, 12CH_2_), 1.43−1.49 (m, 1H in CH_2_), 1.66−1.81 (m, 2H, CH_2_), 1.81−1.96 (m, 1H in CH_2_), 2.40−2.50 (m, 2H, CH_2_), 2.53−2.67 (m, 1H, CH). ^13^C-NMR (101 MHz, D_2_O): *δ* = 14.6, 23.4, 24.5, 27.0, 29.4, 29.6, 29.74, 29.76. 29.79, 30.4, 30.5, 30.6, 30.7, 30.8, 31.1, 31.9, 59.8, 177.7. IR (KBr): 2924.3, 2854.2, 1710.2, 1288.3, 1069.0, 1011.6 cm^−1^. HRMS (ESI): *m/z* calcd for C_18_H_35_O_5_S^−^: 363.2211 [M − H]^−^; found: 363.2205.

## 4. Conclusions

A series of 4-sulfoalkanoic acids with straight C8, C10, C12, C14, C16, and C18 chains was prepared effectively from simple and inexpensive starting materials through the radical addition of methyl 2-((ethoxycarbonothioyl)thio)acetate to linear terminal olefins and subsequent oxidation with peroxyformic acid. The current strategy is a useful and convenient route for the synthesis of dianionic-headed surfactants with a carboxylic acid and sulfonic acid functionalities in the head group region.

## Figures and Tables

**Table 1:** Radical addition of methyl 2-((ethoxycarbonothioyl)thio)acetate **1** with olefins **2**.

Entry	Olefin 2/R	Xanthate 3	Yield (%)
1	*^n^*Bu	**3a**	79
2	*^n^*Hex	**3b**	86
3	*^n^*Oct	**3c**	88
4	*^n^*Decyl	**3d**	86
5	*^n^*Dodecyl	**3e**	80
6	*^n^*Tetradecyl	**3f**	87

**Table 2:** Synthesis of 4-Sulfoalkanoic acids **4**.

Entry	Xanthate 3	R	Acid 4	Yield (%)
1	**3a**	*^n^*Bu	**4a**	99
2	**3b**	*^n^*Hex	**4b**	98
3	**3c**	*^n^*Oct	**4c**	99
4	**3d**	*^n^*Decyl	**4d**	99
5	**3e**	*^n^*Dodecy	**4e**	98
6	**3f**	*^n^*Tetradecyl	**4f**	99

## Supplementary Material

Supplmentary materials are available online. Copies of ^1^H-NMR and ^13^C-NMR spectra of unknown compounds **3** and **4** are included in the Supporting Information.
