# Erratum: Jiang, J.; et al. Expeditious Synthesis of Dianionic-Headed 4-Sulfoalkanoic Acid Surfactants. *Molecules* 2017, *22*, 640

**DOI:** 10.3390/molecules22060877

**Published:** 2017-05-26

**Authors:** Jianhui Jiang, Jiaxi Xu

**Affiliations:** 1 State Key Laboratory of Chemical Resource Engineering, Department of Organic Chemistry, Faculty of Science, Beijing University of Chemical Technology, Beijing 100029, China; xjjjh78@163.com; 2 Engineering Laboratory of Chemical Resources Utilization in South Xinjiang of Xinjiang Production and Construction Corps, College of Life Sciences, Tarim University, Alar, Xinjiang 843300, China

The authors wish to make the following change to their paper [1]. The name of the first author is incorrectly reported to be ‘Jianghui Jiang’. The correct spelling should be ‘Jianhui Jiang’.

We apologize for any inconvenience caused to the readers by this mistake. The manuscript will be updated and the original will remain online on the article webpage.
