# Supplementary material for: Expeditious Synthesis of Dianionic-Headed 4-Sulfoalkanoic Acid Surfactants
Source: Molecules. 2017 Apr 16;22(4):640. doi: 10.3390/molecules22040640 (PMC6154584; doi:10.3390/molecules22040640)

Expeditious synthesis of dianionic headed 4-sulfoalkanoic acid surfactants

Jianhui Jiang and Jiaxi Xu*
State Key Laboratory of Chemical Resource Engineering, Department of Organic Chemistry, Faculty of Science, Beijing University of Chemical Technology, Beijing 100029, People's Republic of China. Tel/Fax +8610-64435565, jxxu@mail.buct.edu.cn.

Contents

Copies of ^{1}H and ^{13}C NMR spectra of products **3** and **4**..S2

Copies of ^{1}H and ^{13}C NMR spectra of products **3** and **4**

3a

^{1}H NMR (400 MHz, $CDCl_3$)

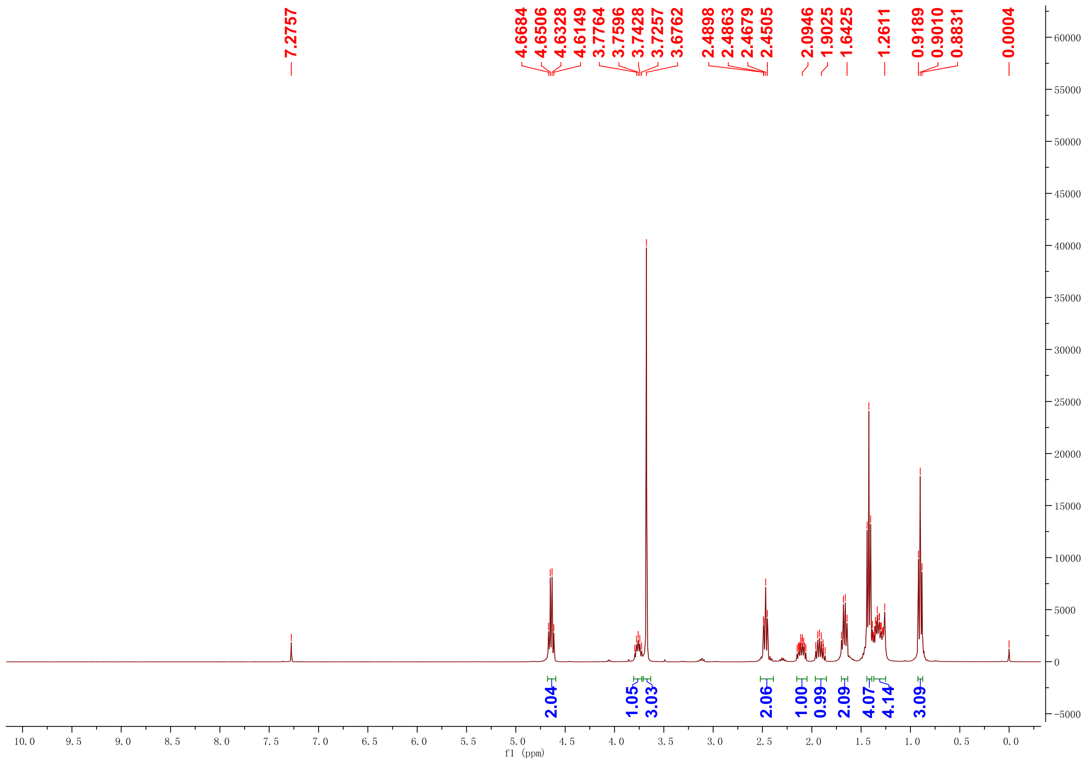

^{13}C NMR (101 MHz, $CDCl_3$)

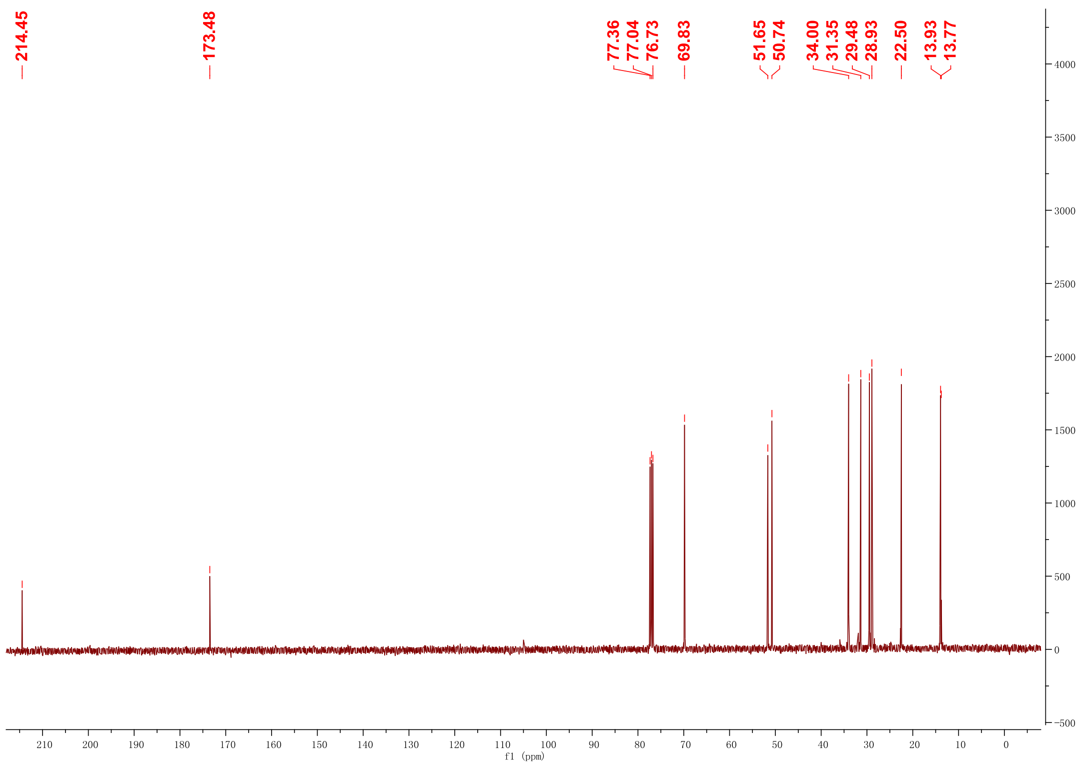

3b

^{1}H NMR (400 MHz, $CDCl_3$)

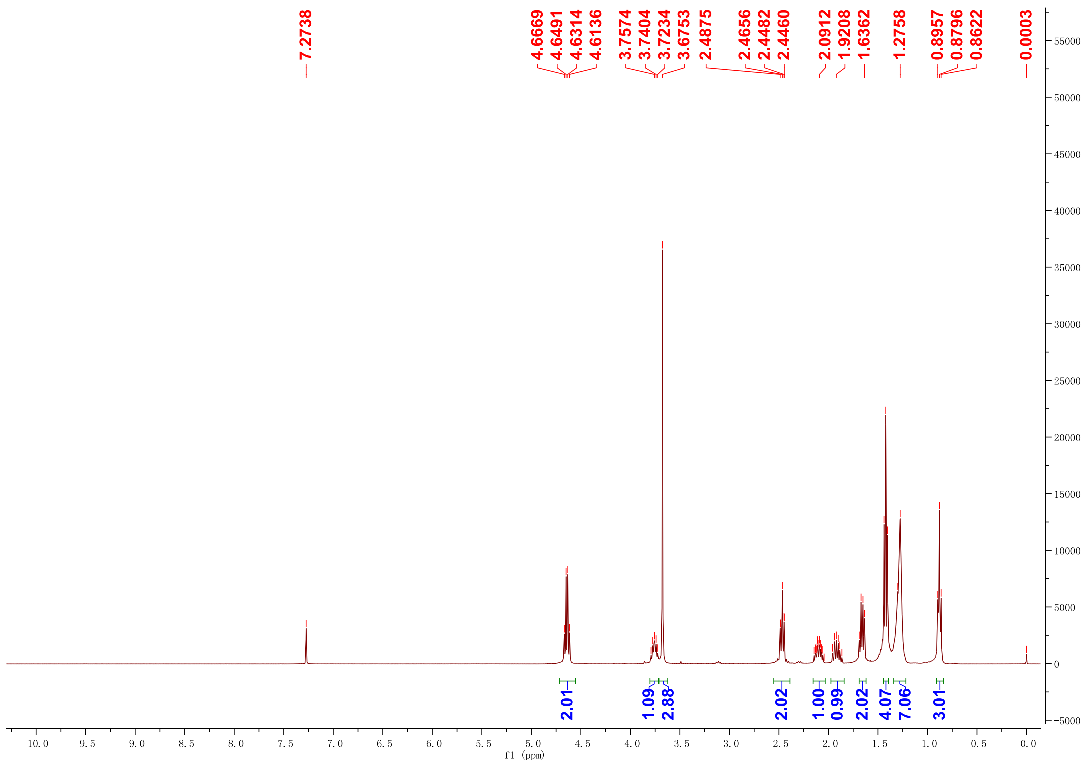

^{13}C NMR (101 MHz, $CDCl_3$)

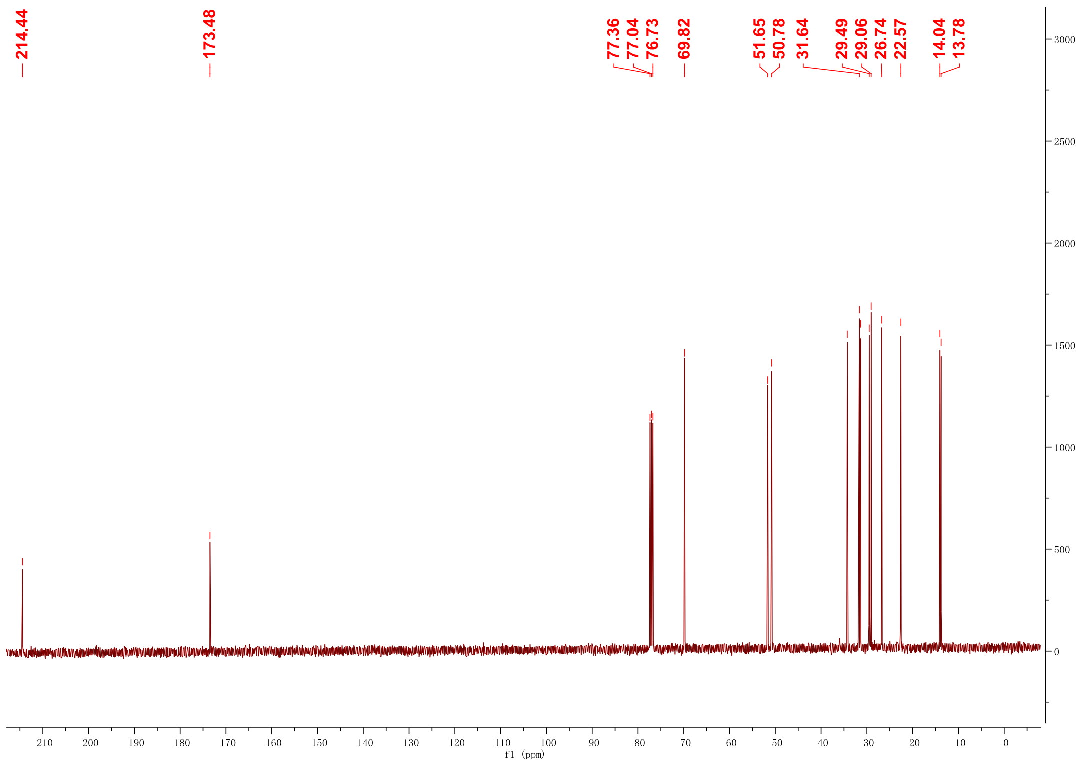

3c

^{1}H NMR (400 MHz, $CDCl_3$)

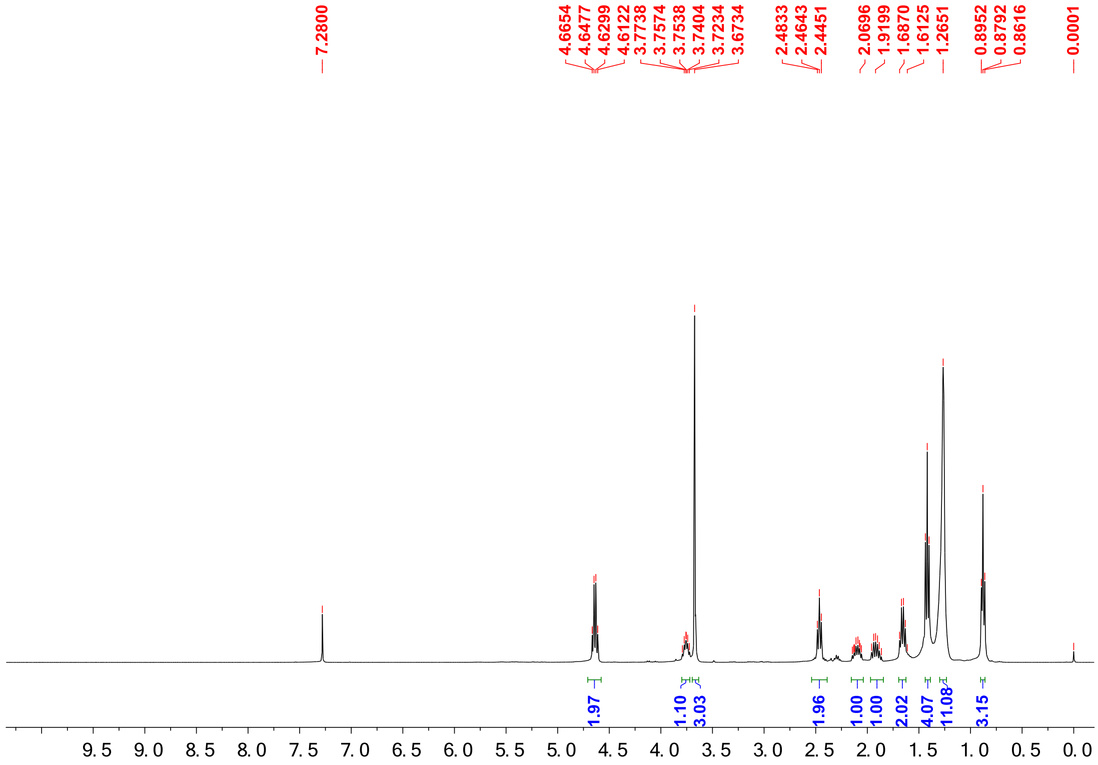

^{13}C NMR (101 MHz, $CDCl_3$)

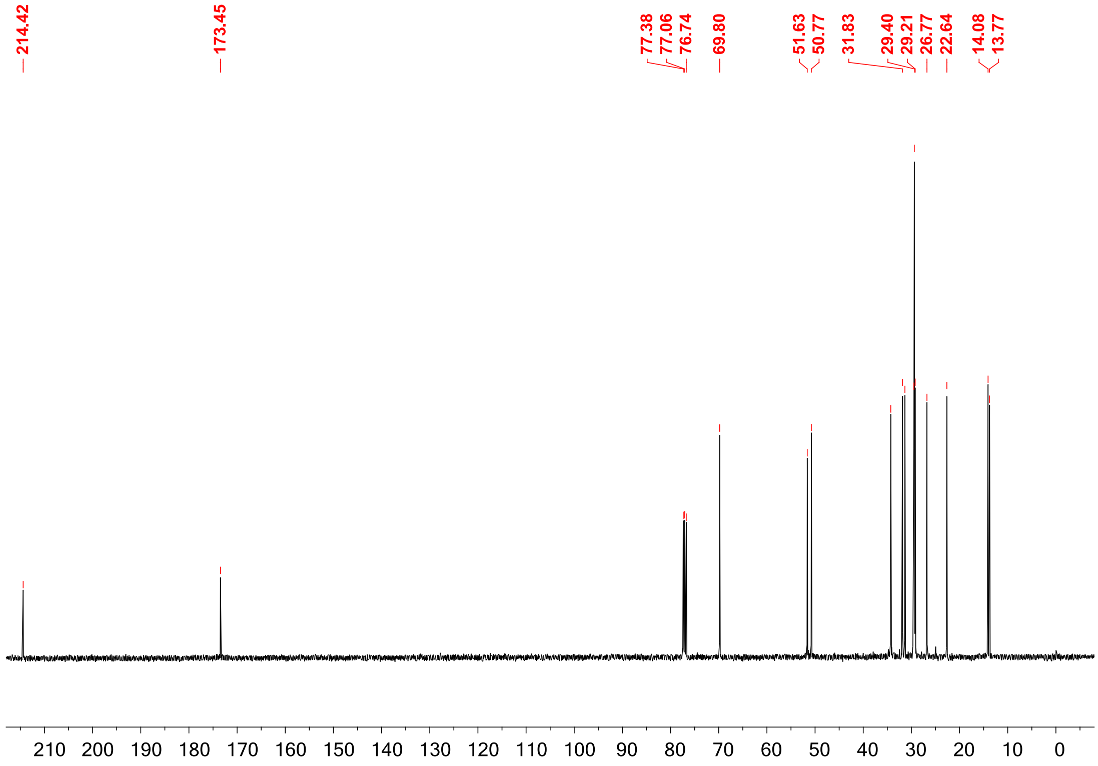

3d

^{1}H NMR (400 MHz, $CDCl_3$)

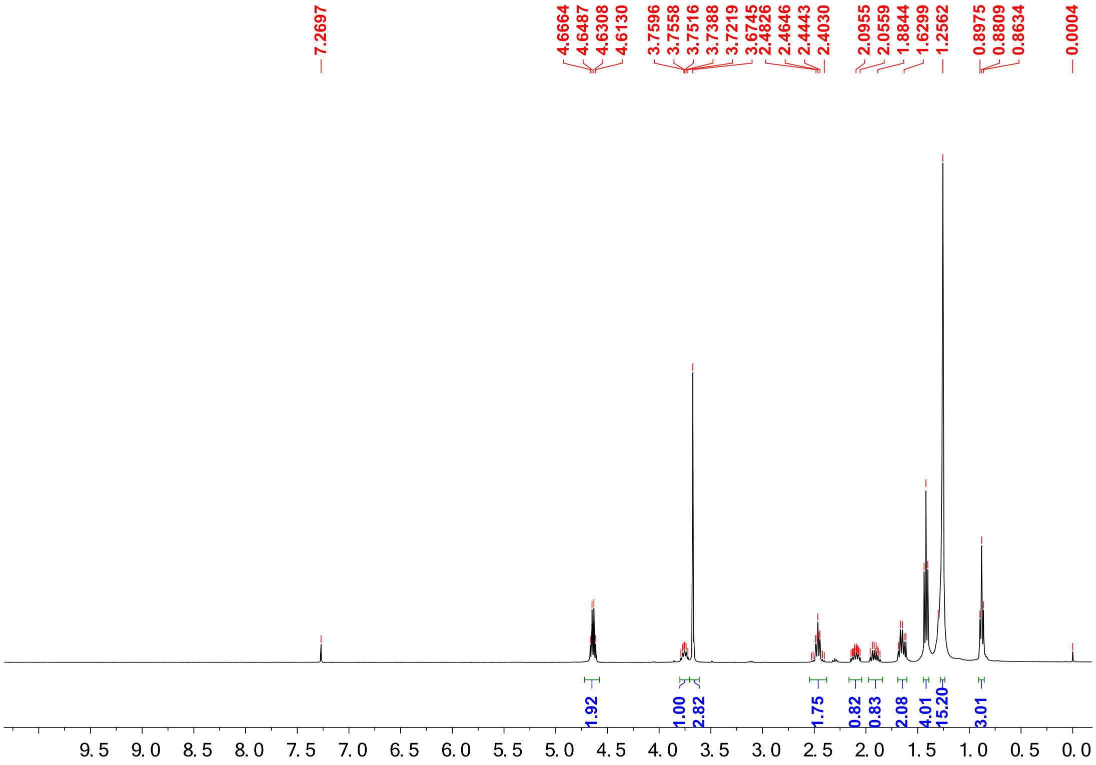

^{13}C NMR (101 MHz, $CDCl_3$)

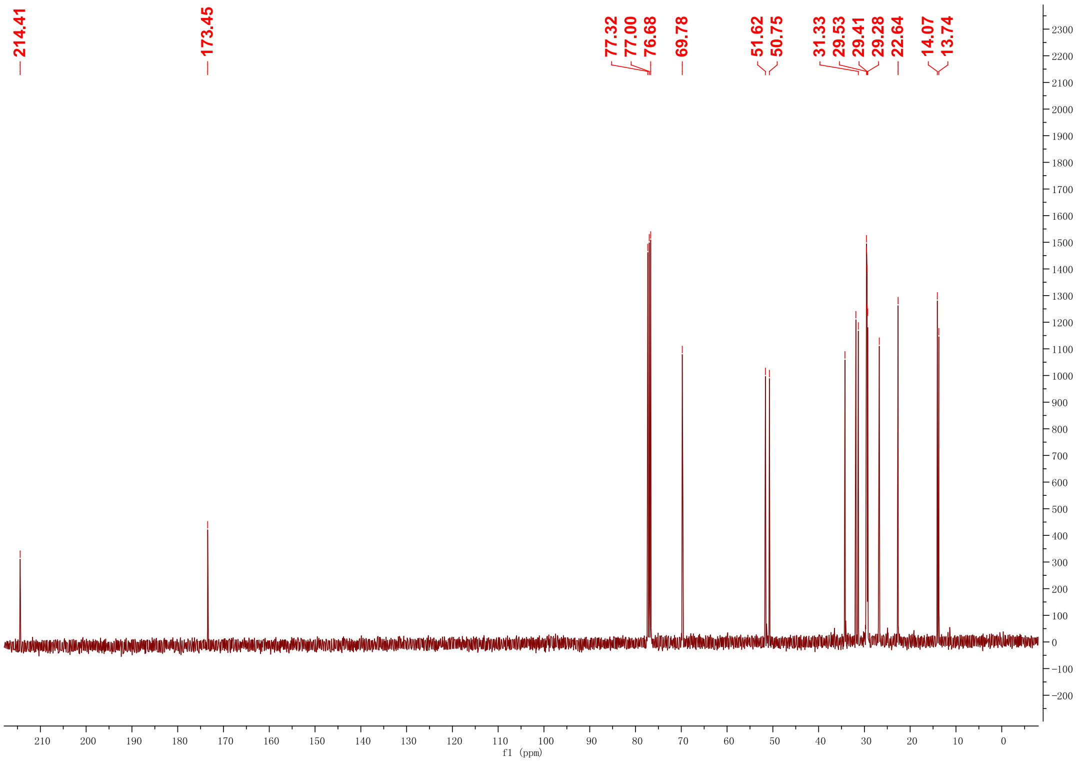

3e

^{1}H NMR (400 MHz, $CDCl_3$)

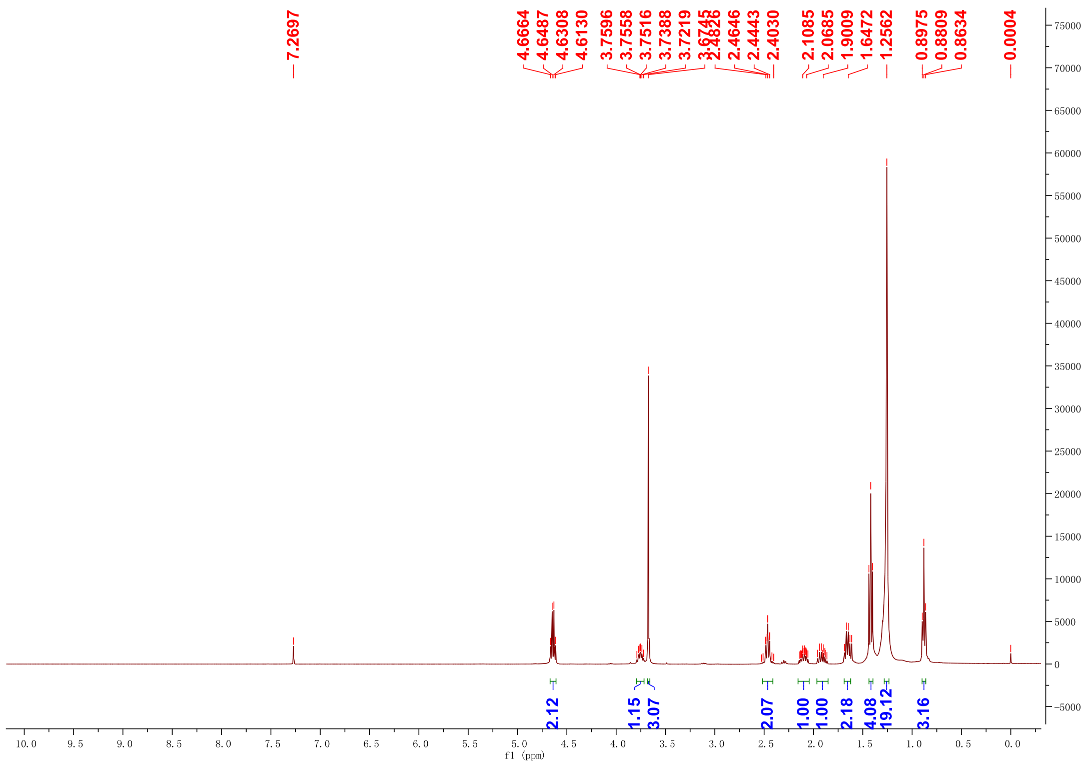

^{13}C NMR (101 MHz, $CDCl_3$)

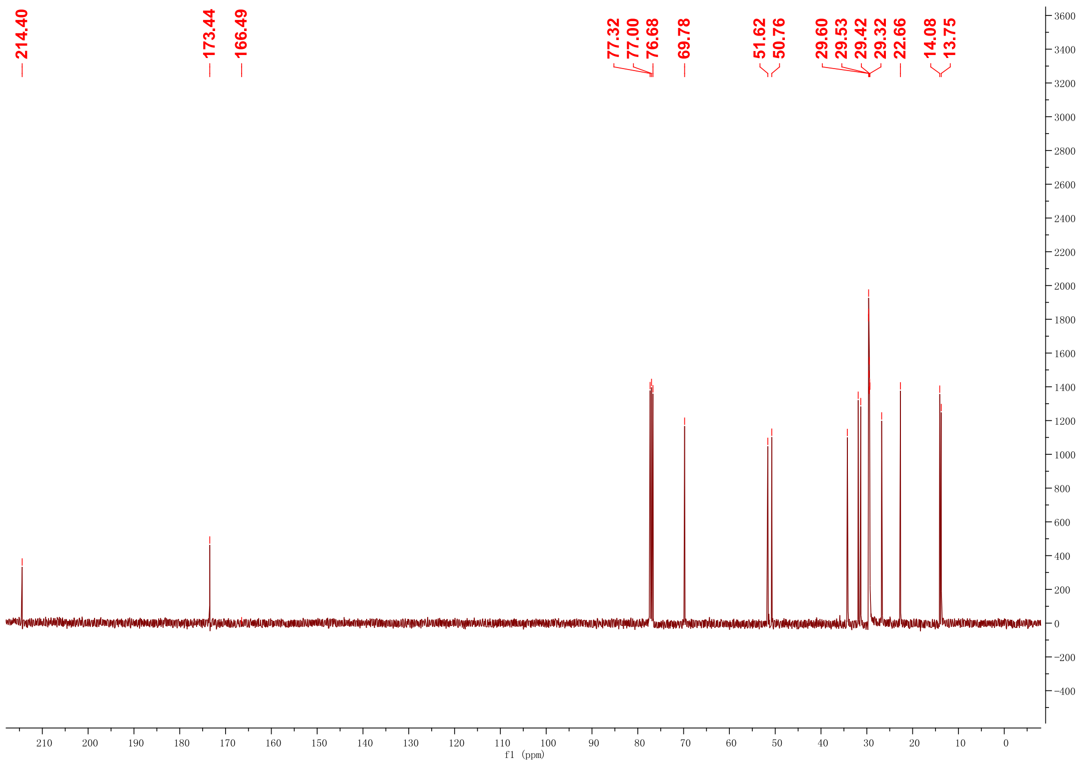

3f

^{1}H NMR (400 MHz, $CDCl_3$)

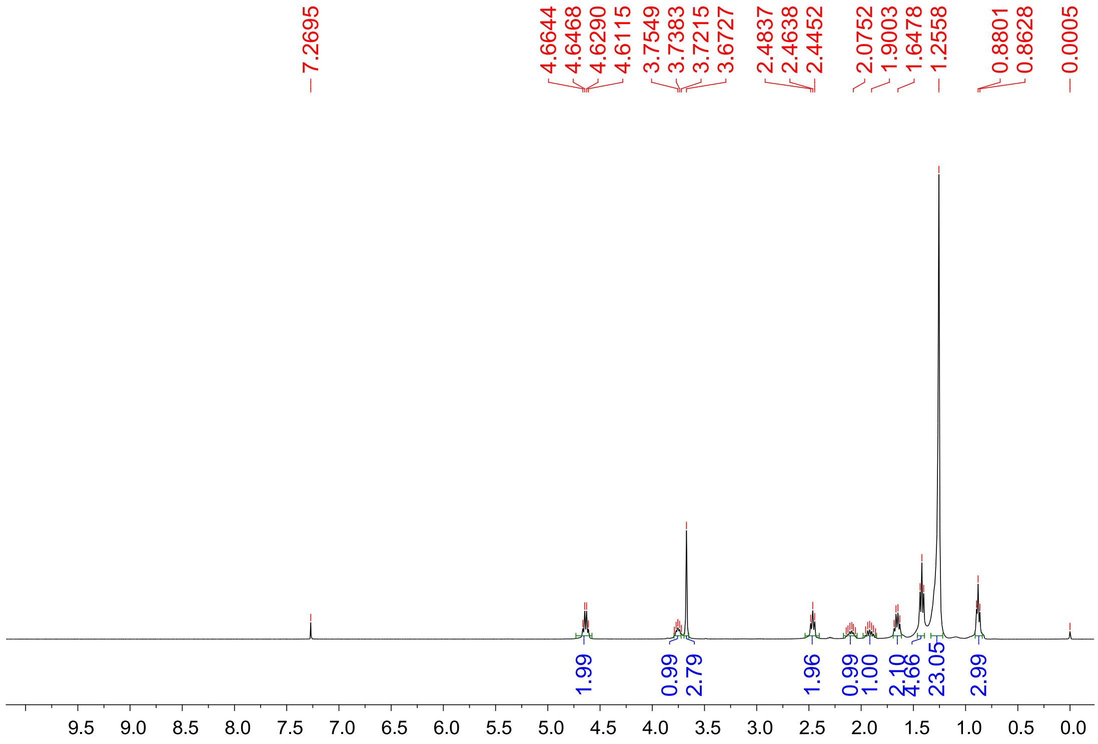

^{13}C NMR (101 MHz, $CDCl_3$)

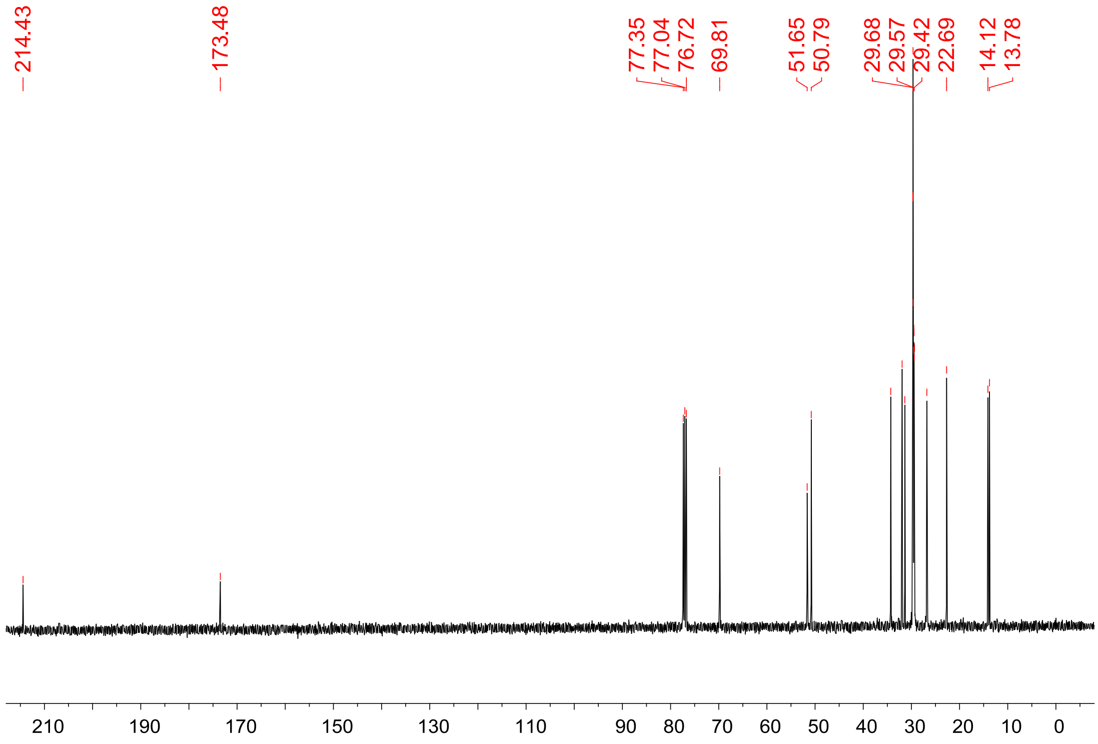

4a

^{1}H NMR (400 MHz, D_2O)

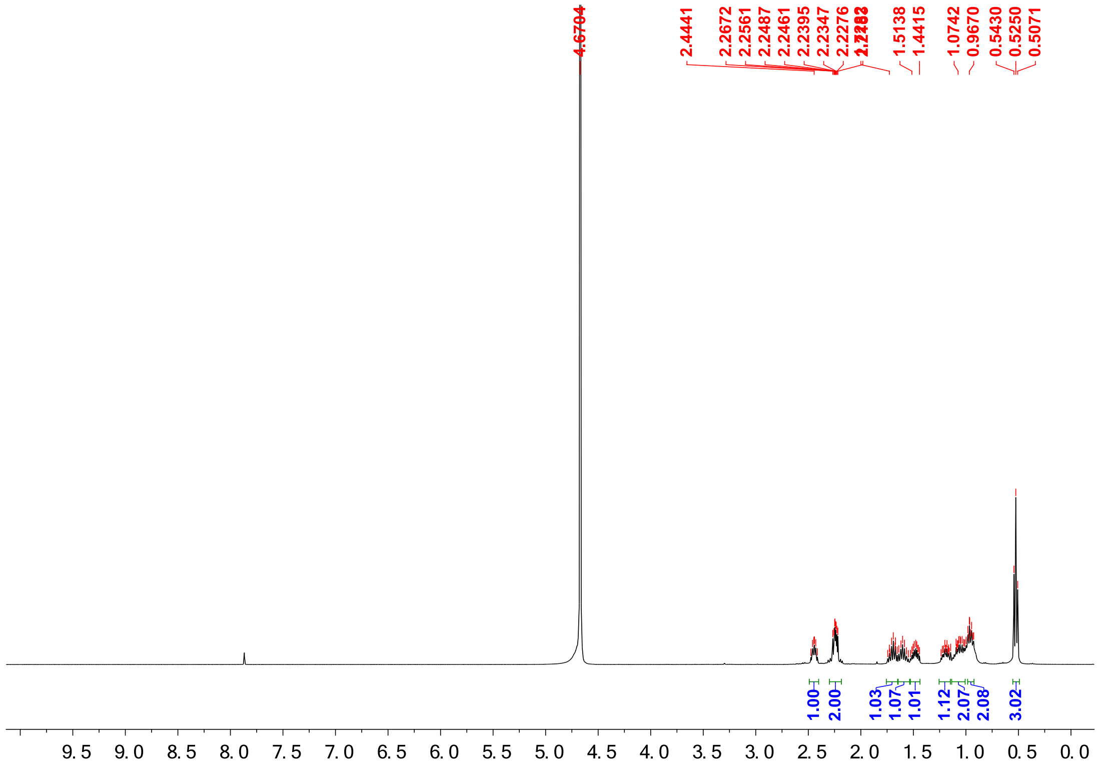

^{13}C NMR (101 MHz, D_2O)

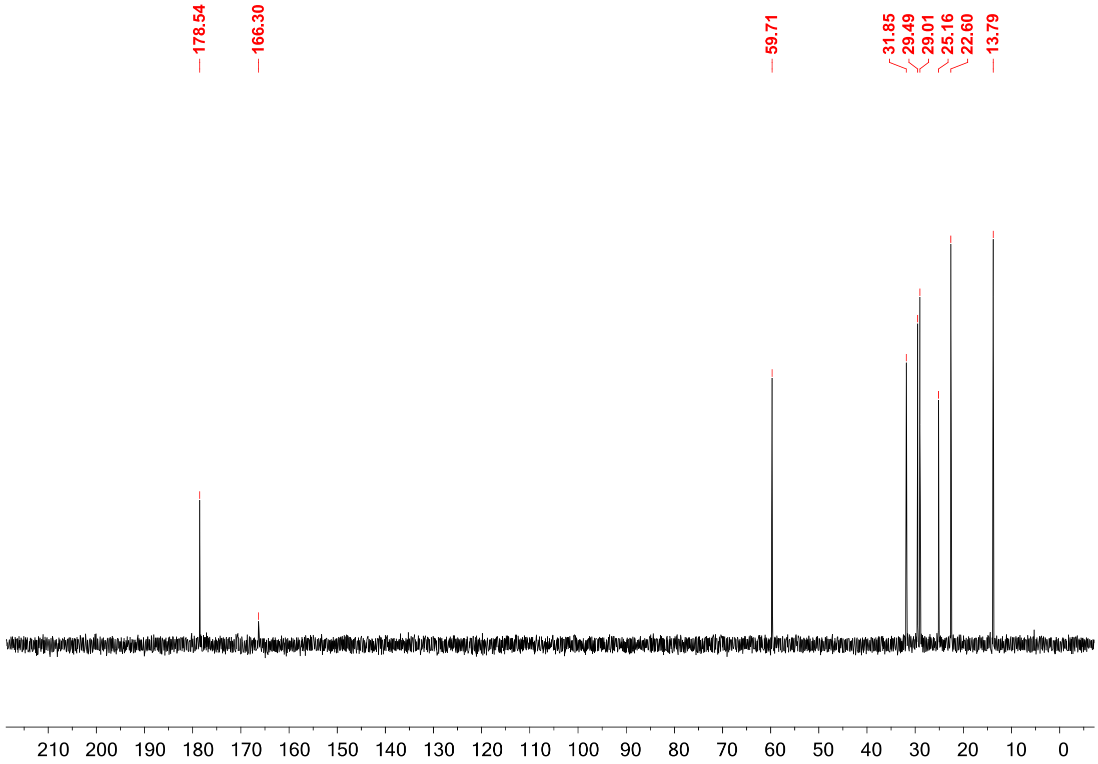

4b

^{1}H NMR (400 MHz, D_2O)

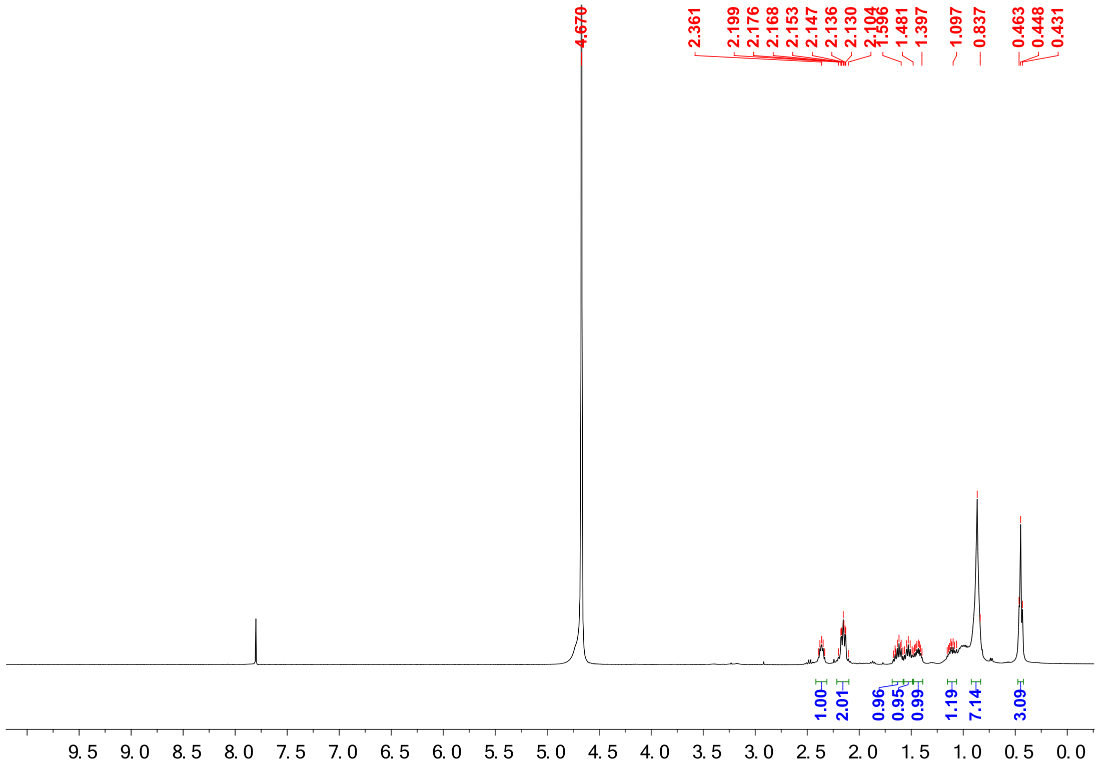

^{13}C NMR (101 MHz, D_2O)

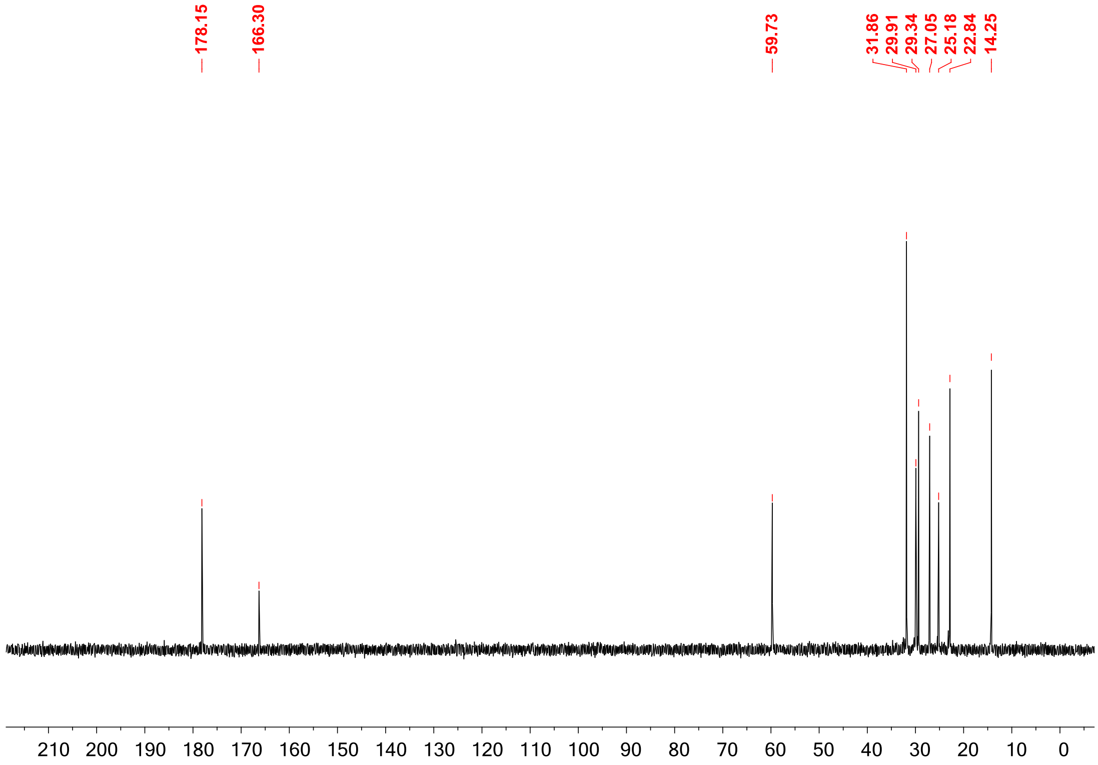

4c

^{1}H NMR (400 MHz, D_2O)

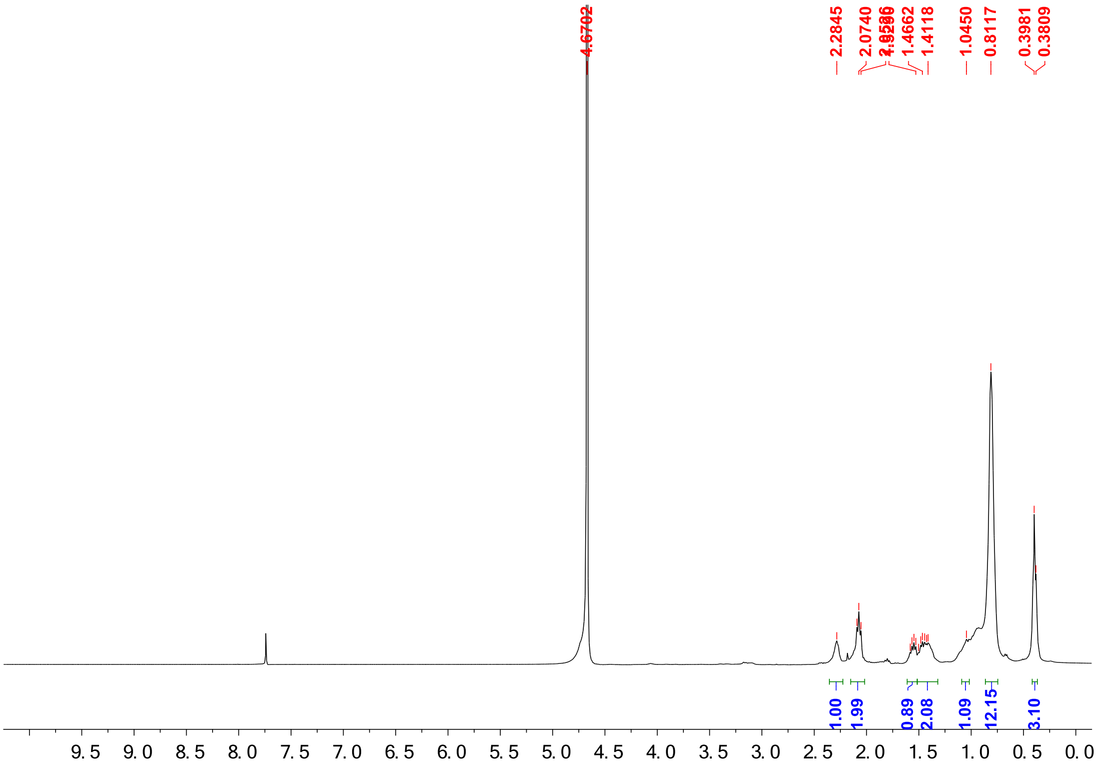

^{13}C NMR (101 MHz, D_2O)

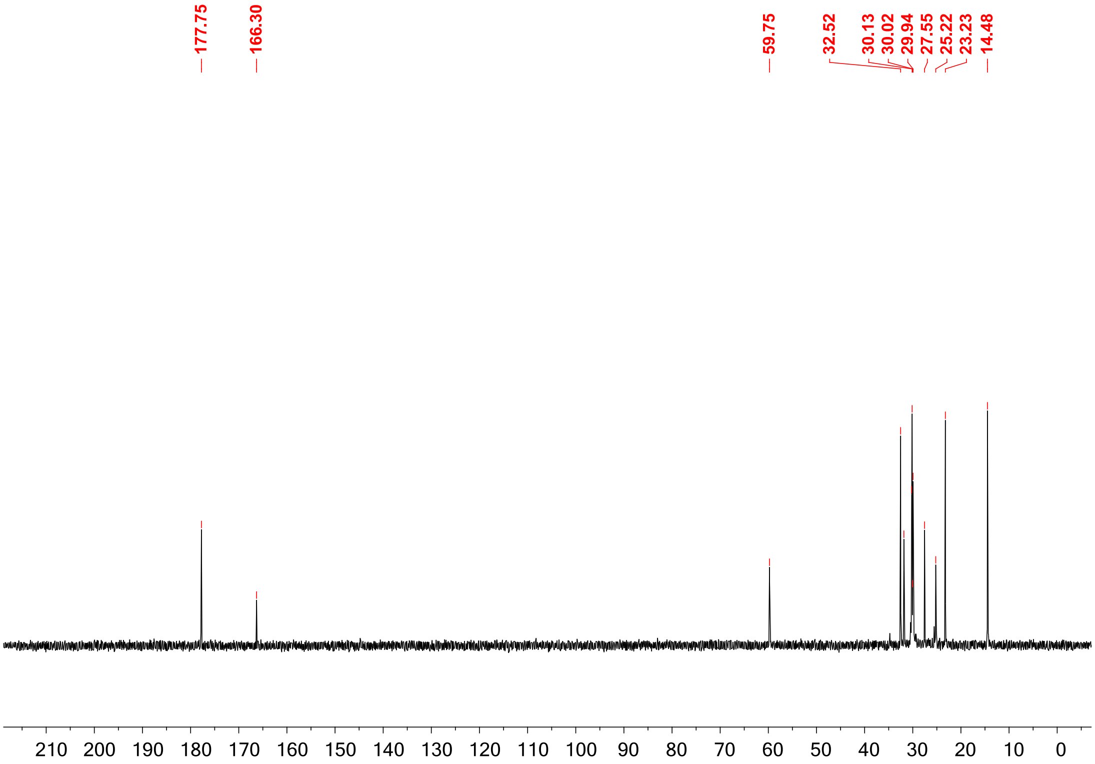

4d

^{1}H NMR (400 MHz, D_2O)

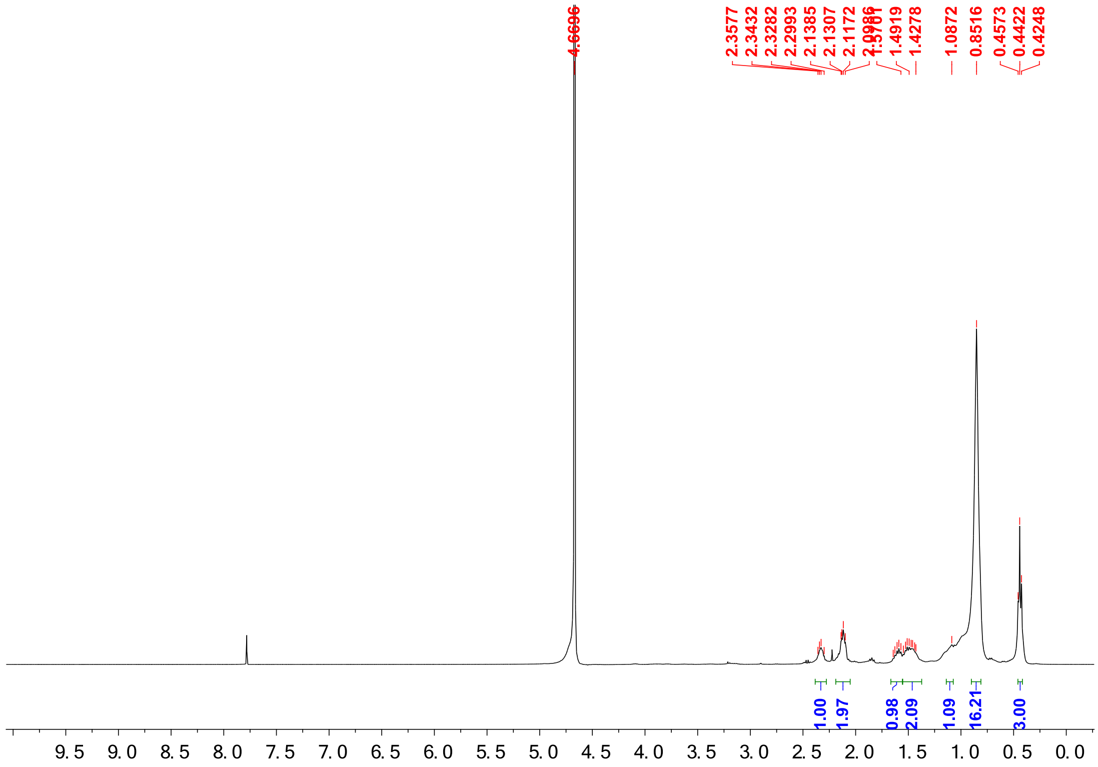

^{13}C NMR (101 MHz, D_2O)

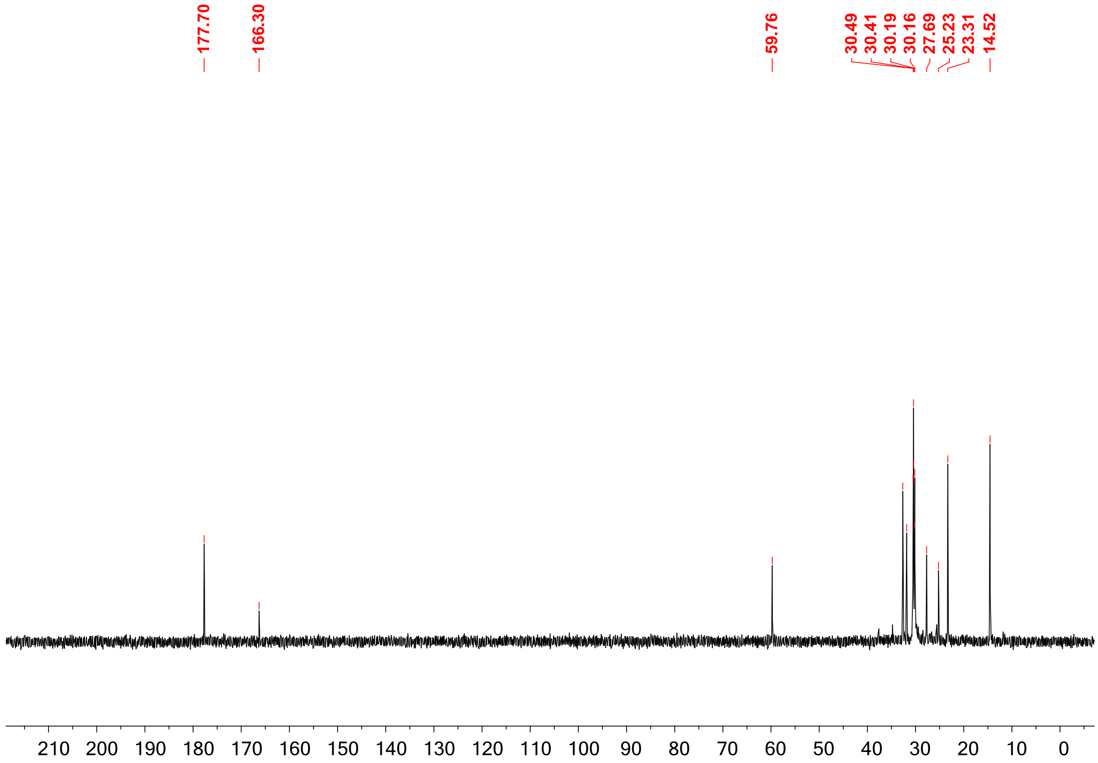

4e

^{1}H NMR (400 MHz, D_2O)

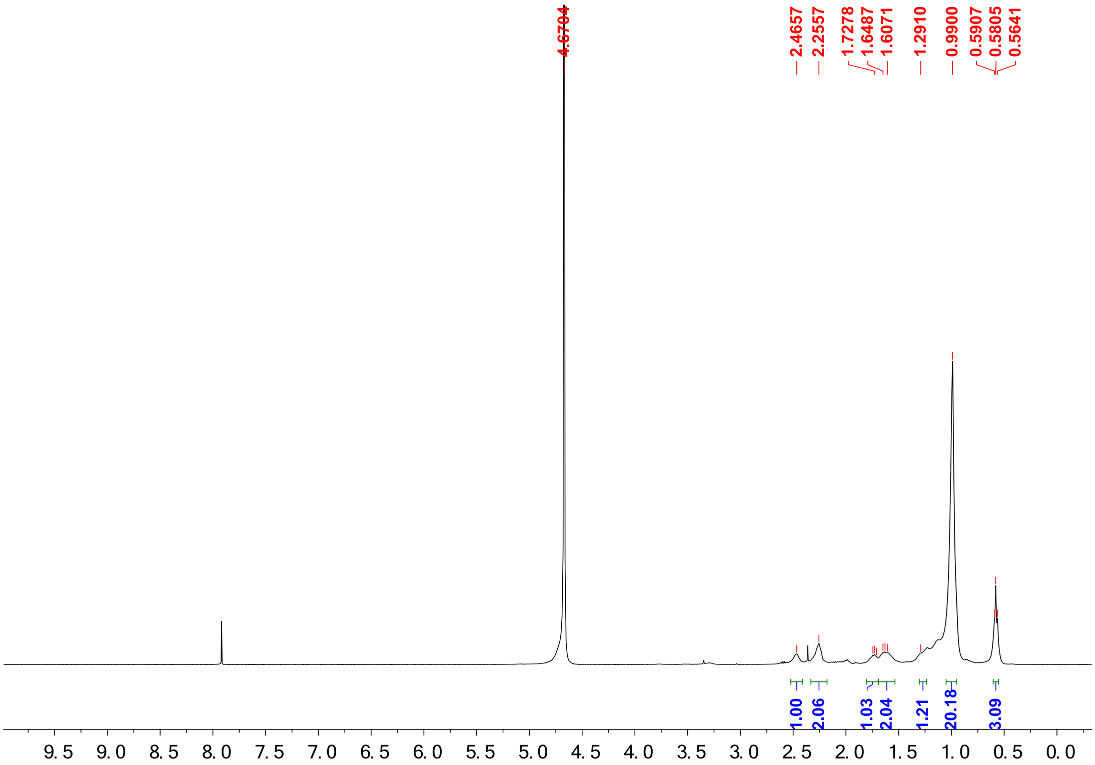

^{13}C NMR (101 MHz, D_2O)

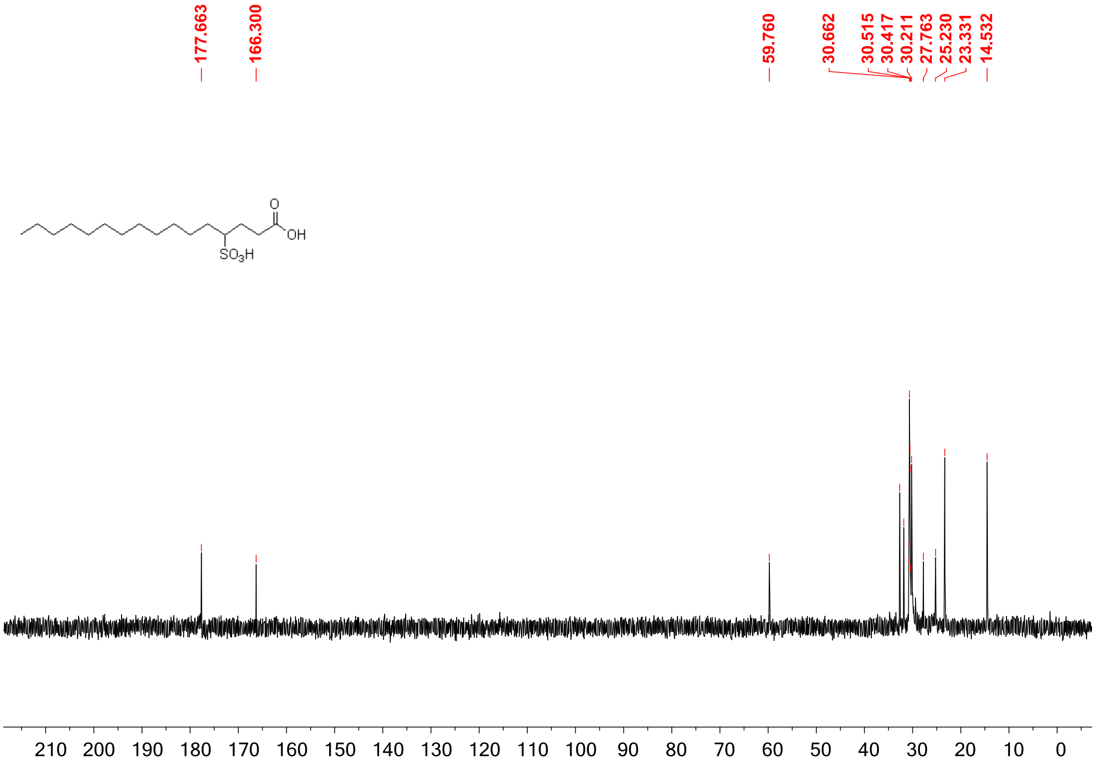

4f

^{1}H NMR (400 MHz, D_2O)

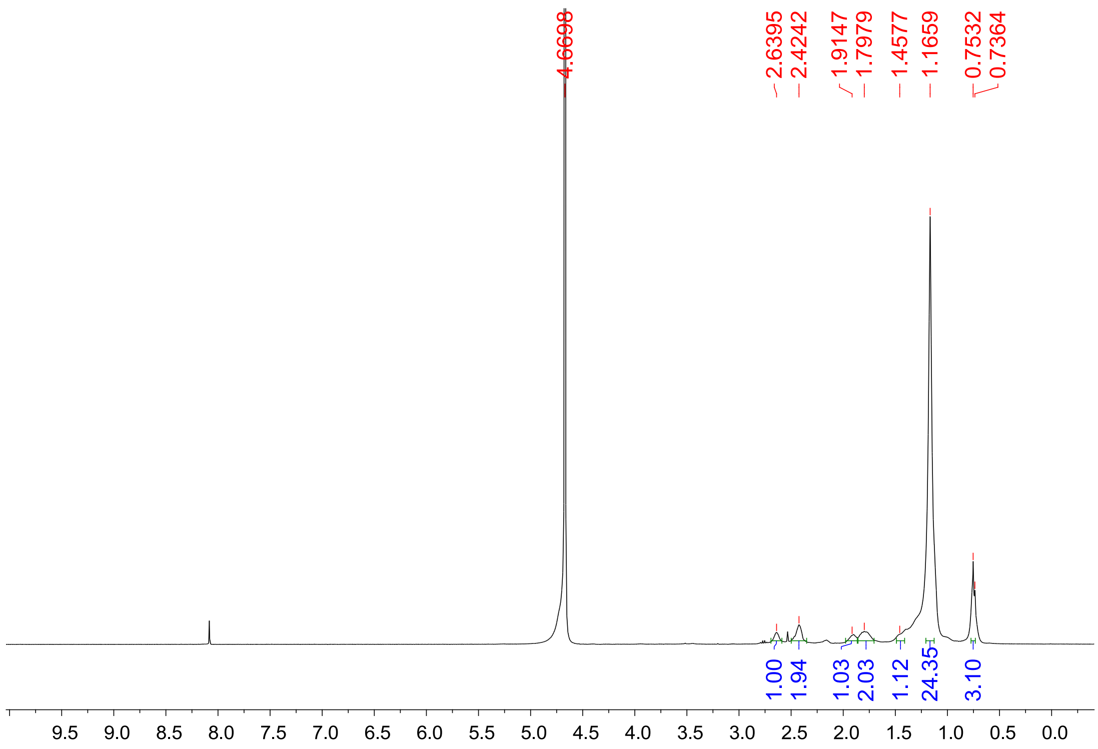

^{13}C NMR (101 MHz, D_2O)

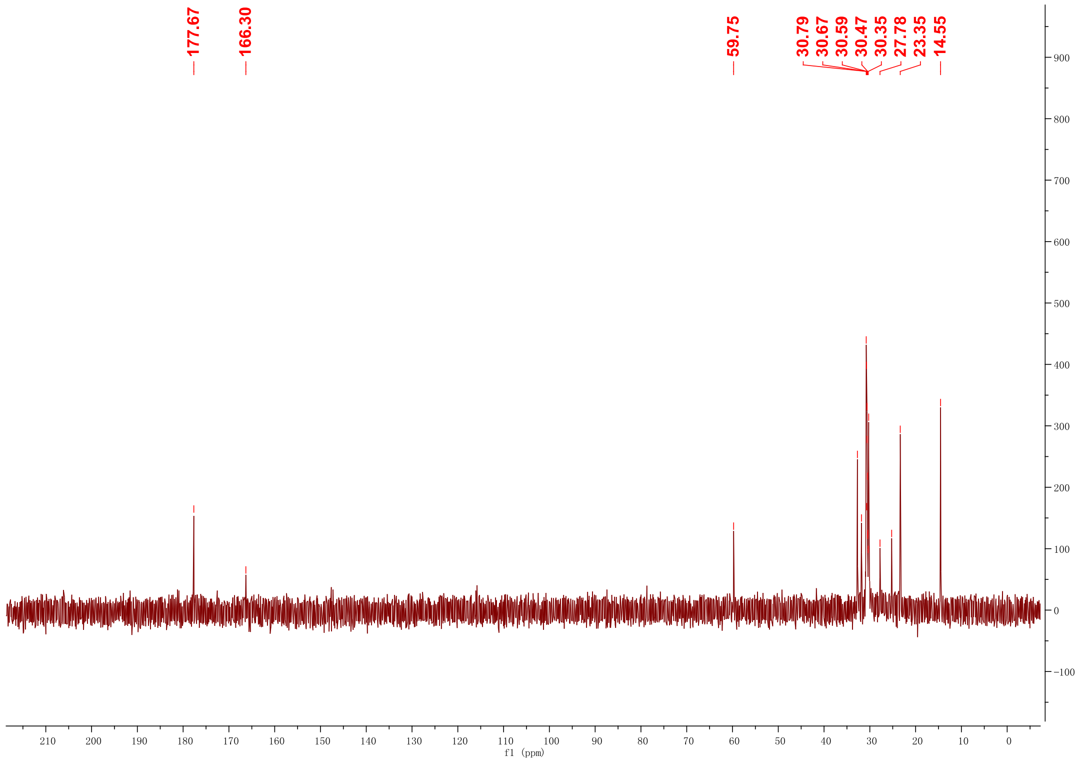

Supplement: Supplementary file 1 [file molecules-22-00640-s001.pdf]
